# Supplementary material for: Wheat VRN1, FUL2 and FUL3 play critical and redundant roles in spikelet development and spike determinacy
Source: Development. 2019 Jul 23;146(14):dev175398. doi: 10.1242/dev.175398 (PMC6679363; doi:10.1242/dev.175398)
Supplement: Supplementary information [file develop-146-175398-s1.pdf]

SUPPLEMENTARY TABLES AND FIGURES

Supplementary Tables

Table S1. Primers used for genotyping the *ful2*, *ful3, vrn1* and *vrn2*-null mutants

Mutant		Primer sequences	Marker type and restriction enzyme
ful-3A	Forward	ATGGATGTGATTCTTGAAC	CAPS (*Sml*I)
	Reverse	AAACGTAAATACAGTGGAAC	
ful-3B	Forward	GAAGAGTCAAAGGTATTGTATTTCTA	dCAPS (*Pvu*II)
	Reverse	TGCTTCAAAGAACTATCCAGCT	
ful-2A	Forward	CACATTACCTTACCATCTACTCAC	CAPS (*HpyA*V)
	Reverse	CATGCAACAGAATTATACAGC	
ful-2B	Forward	CCATTGACCATCAACTAGATCA	CAPS (*Btsα*I)
	Reverse	TGAACCAGCATTCCTGATA	
vrn-1A	Forward	GCCTATTTGTAGCATTTCTGTCATT	CAPS (*Bsg*I) [1]
	Reverse	GAACATCTCAGTCTAGAATCTGAT	
vrn-1B	Forward	CGCCTCACCCAACCACCTGAC	CAPS (*Bsl*I) [1]
	Reverse	ACGGATGGAAACAGCTACCGA	
vrn2	Forward	AACGCTTTATGATGCCAAGG	CAPS for linked *SNF2*(*HpyCh4*IV) [2]
	Reverse	TGTGGACAGAACTGGTTTGC	

[1] **Chen, A. and Dubcovsky, J.** (2012). *PLoS Genetics* **8**, e1003134.

[2] **Distelfeld, A., Tranquilli, G., Li, C., Yan, L. and Dubcovsky, J.** (2009). *Plant Physiology* **149**, 245-257.

Development • Supplementary information

Table S2. Primers used the real time Q-PCR experiments.

Gene		Primer sequence	Primer efficiency
VRT2	Forward	AGCAGCCGGCTATTGATTTA	101%
	Reverse	TTCCATCTGCTGCAACTCAC	
BM1	Forward	CCAGCAGATGAGAGGAGAGG	100%
	Reverse	GGCTCTTGGTTTTCAGAACG	
BM10	Forward	CACAGGGTGCTTCAGACAAA	103%
	Reverse	TTGGAATCTGGCCTACTTGG	
CEN-2	Forward	CTCGTCGTTGGAAAGGTCAT	105%
	Reverse	TGAACACCTGCTTGTTGGAG	
CEN-4	Forward	TCGTCGTCGGAAAGGTCATC	100%
	Reverse	GAACTCCTGGCCATTGGACA	
CEN-5	Forward	GGCCATGAGCTCTACCCATC	107%
	Reverse	ACCCCCTTGGACCTCTACTC	
FT1	Forward	CAGCAGCCCAGGGTTGAG	100% [1]
	Reverse	ATCTGGGTCTACCATCACGAGTG	

[1] **Yan L, Fu D, Li C, Blechl A, Tranquilli G, Bonafede M, Sanchez A, Valarik M, Yasuda S, Dubcovsky J.** (2006) PNAS **103**:19581-6.

Development • Supplementary information

Table S3. Three-way ANOVA for heading time. Population segregating for *VRN1* (*vrn1*-null vs. heterozygous *Vrn1*), *VRN2* (*vrn2*-null vs. wild type *Vrn2*) and *FUL2* (*Ubi::FUL2* versus non-transgenic control). The population was generated from the cross between Kronos *vrn1vrn2*-null and Kronos overexpressing *FUL2* under the maize *UBIQUITIN* promoter (*Vrn1Vrn2*). Normality of residuals was confirmed by the Shapiro-Wilk test ($P = 0.3516$).

Dependent Variable: HD

Source	DF	Sum of Squares	Mean Square	F Value	Pr > F
Model	7	19506.2	2786.6	311.93	<.0001
Error	47	419.9	8.9		
Corrected Total	54	19926.1			

R^2= 0.978929

Source	DF	Type III SS	Mean Sq.	F Value	Pr > F
VRN1	1	7339.3	7339.3	821.57	<.0001
FUL2	1	3336.2	3336.2	373.46	<.0001
VRN1*FUL2	1	1502.7	1502.7	168.21	<.0001
VRN2	1	13441.2	13441.2	1504.61	<.0001
VRN1*VRN2	1	2366.2	2366.2	264.88	<.0001
FUL2*VRN2	1	1696.9	1696.9	189.95	<.0001
VRN1*FUL2*VRN2	1	717.9	717.9	80.36	<.0001

Development • Supplementary information

Supplementary Figures

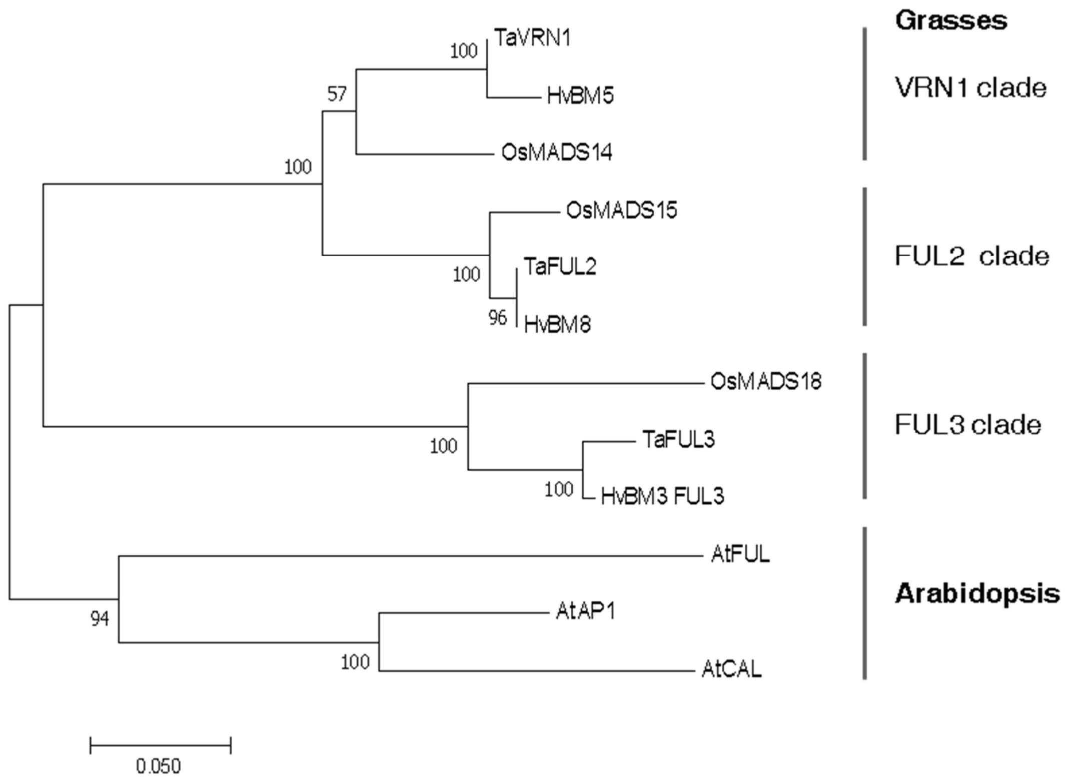

Fig. S1. Phylogeny of duplicated Arabidopsis AP1/CAL/FUL and grasses VRN1/FUL2/FUL3 clades. Evolutionary history inferred using the Neighbor-Joining method (Saitu and Nei, 1987). The optimal tree is shown with bootstrap values >50 shown in the nodes (based on 1000 replicates, Felsenstein, 1985). The tree is drawn to scale, with branch lengths in the same units as those of the evolutionary distances used to infer the phylogenetic tree. The evolutionary distances were computed using the Poisson correction method (Zuckerkandl and Pauling, 1965) and are in the units of the number of amino acid substitutions per site. All positions containing gaps and missing data were eliminated. There were a total of 176 positions in the final dataset. Evolutionary analyses were conducted in MEGA7 (Kumar et al. 2016).). The duplication that originated the VRN1 and FUL2 clusters occurred close to the base of the grass divergence, whereas the duplication that originated the FUL3 clade occurred close to the base of the monocots (Preston and Kellogg, 2006). At = *Arabidopsis thaliana*, Os = *Oryza sativa*, Hv = *Hordeum vulgare*, Ta = *Triticum aestivum.*

References for Fig. S1.

Felsenstein J. (1985). *Evolution* **39**,783-791.

Kumar S., Stecher G., and Tamura K. (2016). *Molecular Biology and Evolution 33*, 1870-1874.

Preston, J. C. and Kellogg, E. A. (2006). *Genetics* **174**, 421-437.

Saitou N. and Nei M. (1987). *Molecular Biology and Evolution* **4**,406-425.

Zuckerkandl E. and Pauling L. (1965). Ed. by V. Bryson and H.J. Vogel, pp. 97-166. Acad. Press, New York.

Development • Supplementary information

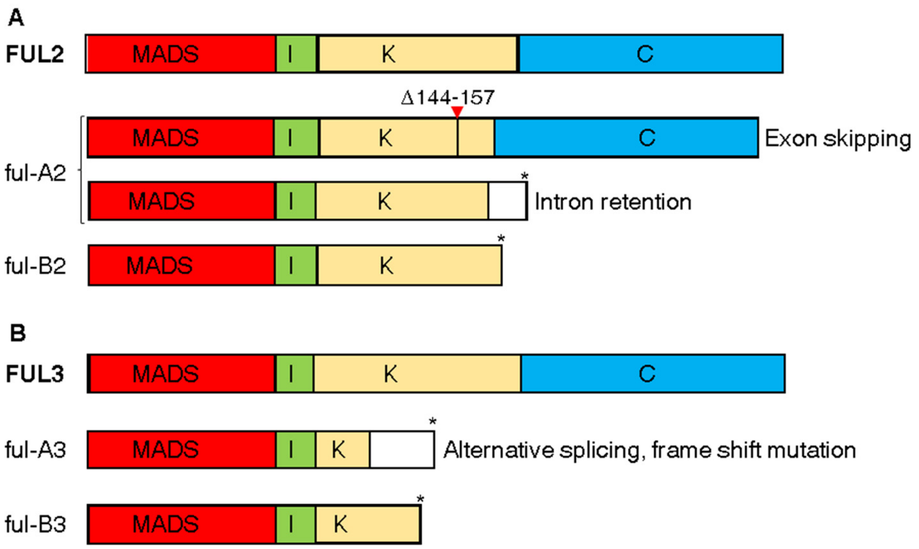

Fig. S2. Selected *ful2* and *ful3* mutations and their effect on the encoded proteins. Wild type FUL2 and FUL3 MADS-box proteins showing conserved domains are included as reference. MADS= MADS box domain, I= intervening domain, K = keratin-like box domain, C = C-terminal domain. (A) *ful-A2* mutation in the splice donor site of the 5th intron results in two alternative splice forms. The first one skips the 5th exon generating a deletion of 14 amino acids in the K-box (Δ144-157) and the second one retains the 5th intron resulting in altered translation (white rectangle) and a premature stop codon. The *ful-B2* mutation generates a premature stop codon (Q162*). (B) The *ful-A3* mutation in the splice acceptor site of the 3rd intron shifts the reading frame by one nucleotide generating a premature stop codon. The *ful-B3* mutation generates a premature stop codon (Q132*). More detailed descriptions are presented in Material and Methods.

Development • Supplementary information

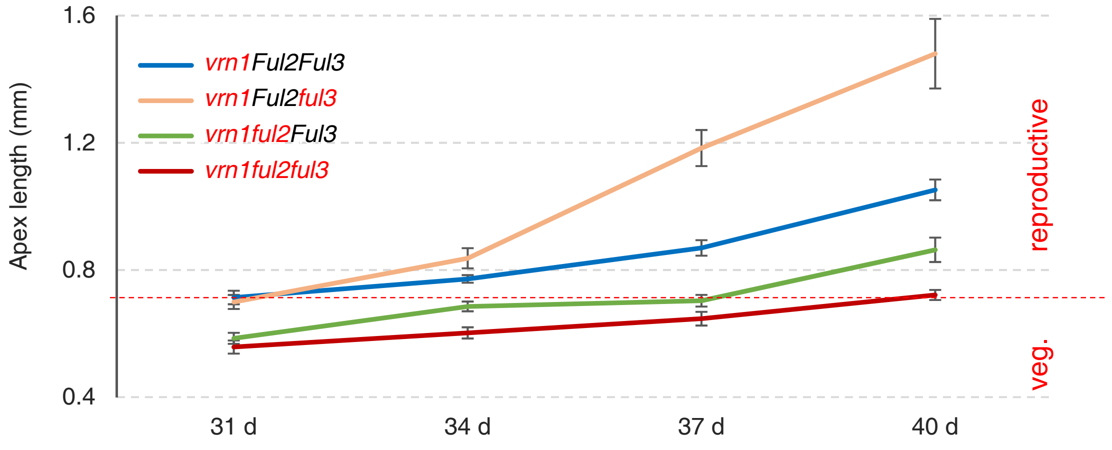

Fig. S3. Time course of shoot apical meristem (SAM) elongation and transition to the reproductive stage in *ful2* and *ful3* mutants in a *vrn1*-null background. The length of the SAM was measured every 3 d from day 31 when we observed elongation in the *vrn1Ful2Ful3* control. The red dotted line indicates the transition of the SAM to the reproductive stage. Six SAMs were measured per time point genotype combination. Error bars are standard errors of the means.

Development • Supplementary information

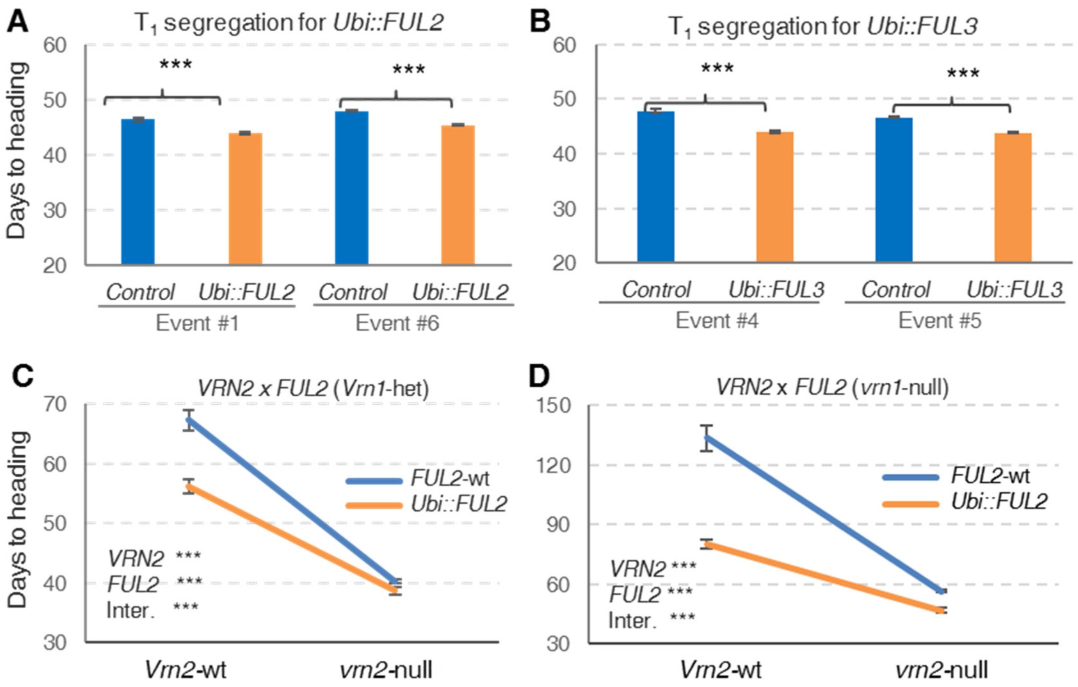

Fig. S4. **Effect of *Ubi::FUL2* and *Ubi::FUL3* on heading time**. (A-B) Heading time of Kronos T_1 transgenic plants from two independent events segregating for (A) *FUL2* (*Ubi::FUL2*, n= 4-23) and (B) *FUL3* (*Ubi::FUL3*, n= 9-31). (C-D) Two way interactions for F_2 plants segregating for *VRN1, VRN2* and *FUL2*. (C) *VRN2 x FUL2* in a *Vrn1* heterozygous background (for both genomes). (D) *VRN2 x FUL2* in a *vrn1*-null background. *P* values correspond to a 2 x 2 factorial ANOVA (3-way ANOVA in Table S4). Error bars are SEM. *** = $P < 0.0001$, NS = $P > 0.05$.

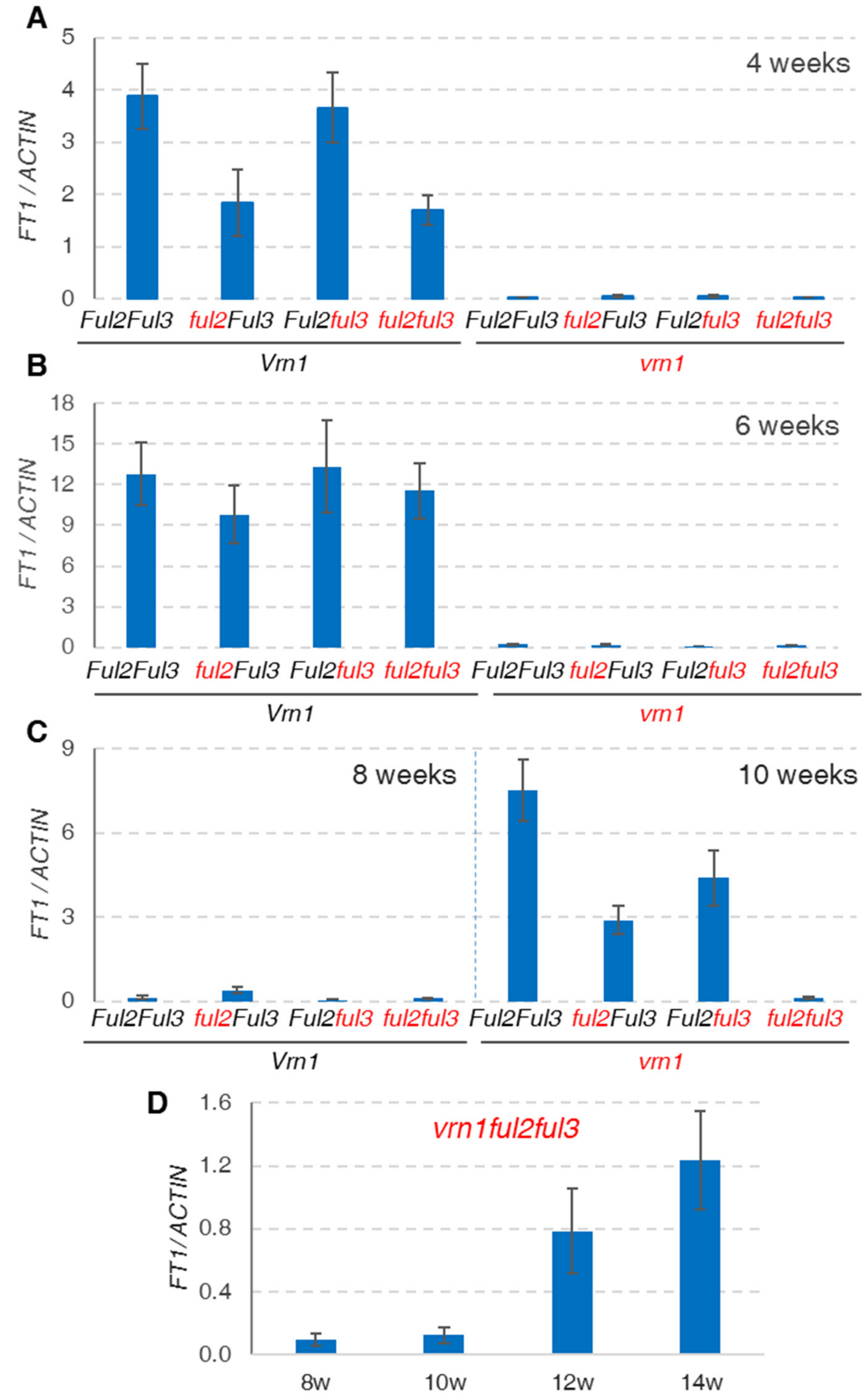

Fig. S5. *FT1* transcript levels in leaves. (A-B) Eight homozygous combinations of *VRN1*, *FUL2* and *FUL3* (mutant alleles in red). (A) 4-week-old plants. (C) 8- and 10-week-old plants (only *vrn1*-null genotypes). (D) Triple *vrn1ful2ful3*-null mutant 8- to 14-week-old plants. All these mutants are in a Kronos *vrn2*-null background. Transcript levels were calculated relative to the *ACTIN* as endogenous control using the ΔCt method (scales are comparable across graphs).

Development • Supplementary information

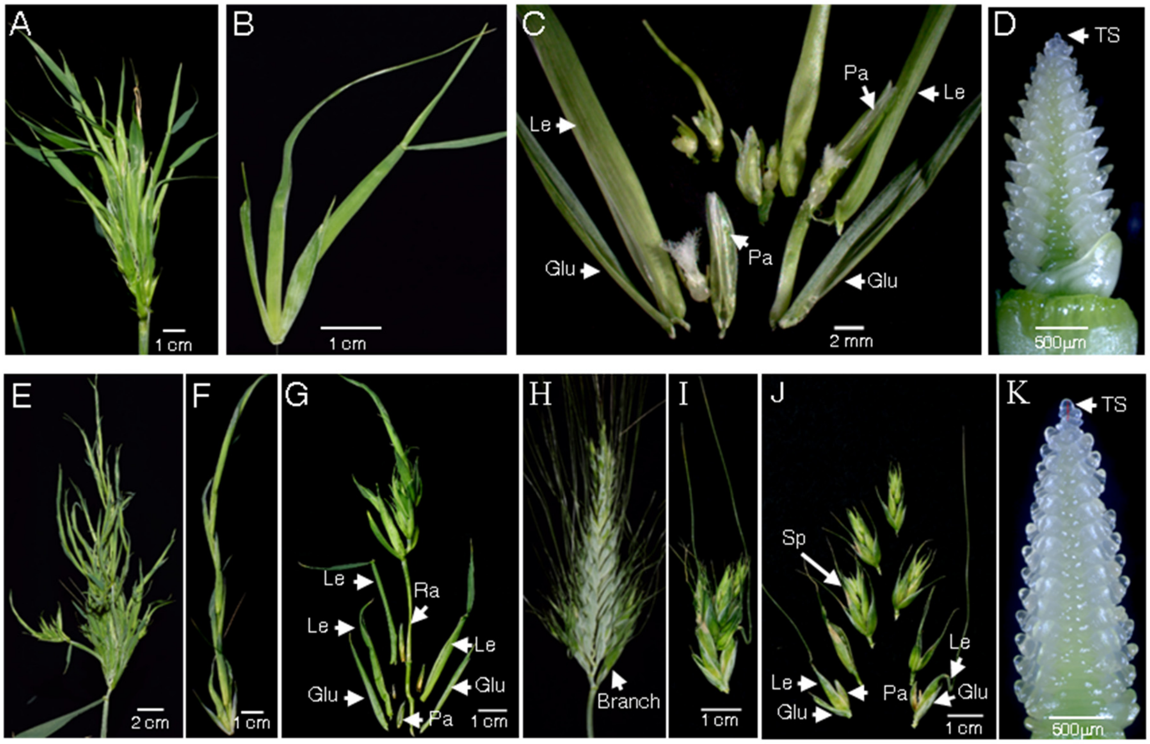

Fig. S6. **Phenotypic characterization of heterozygous mutants containing one copy of *VRN1* or *FUL2***. (A-C) *ful2*-null/*Vrn-A1 vrn-B1*-null. (A) "Head" at early stage. (B) Detached spikelet. (C) Dissection of an individual spikelet showing floral organs including lodicules, ovaries and stamens. (D-G) *ful2*-null/*vrn-A1*-null *vrn-B1* (functional winter allele). (D) Terminal spikelet (TS) confirming spike meristem determinacy. (E) Head at later stage. (F) Detached "spikelet" showing indeterminate growth. (G) Spikelet dissection showing rachilla elongation. (H-K) *vrn1*-null/*ful-A2*-null *Ful-B2* (produces viable grain). (H) Representative spikes showing formation of lateral branches in the basal region of the spike and extra florets in the terminal spikelet. (I) Detached lateral branch. (J) Dissection of the lateral branch. (K) Terminal spikelet (TS) confirming spike meristem determinacy. Sp= spikelets, Glu= glumes, Pa= palea, Le= lemma, Ra= rachilla. All these mutants are in a Kronos *vrn2*-null background.

Development • Supplementary information

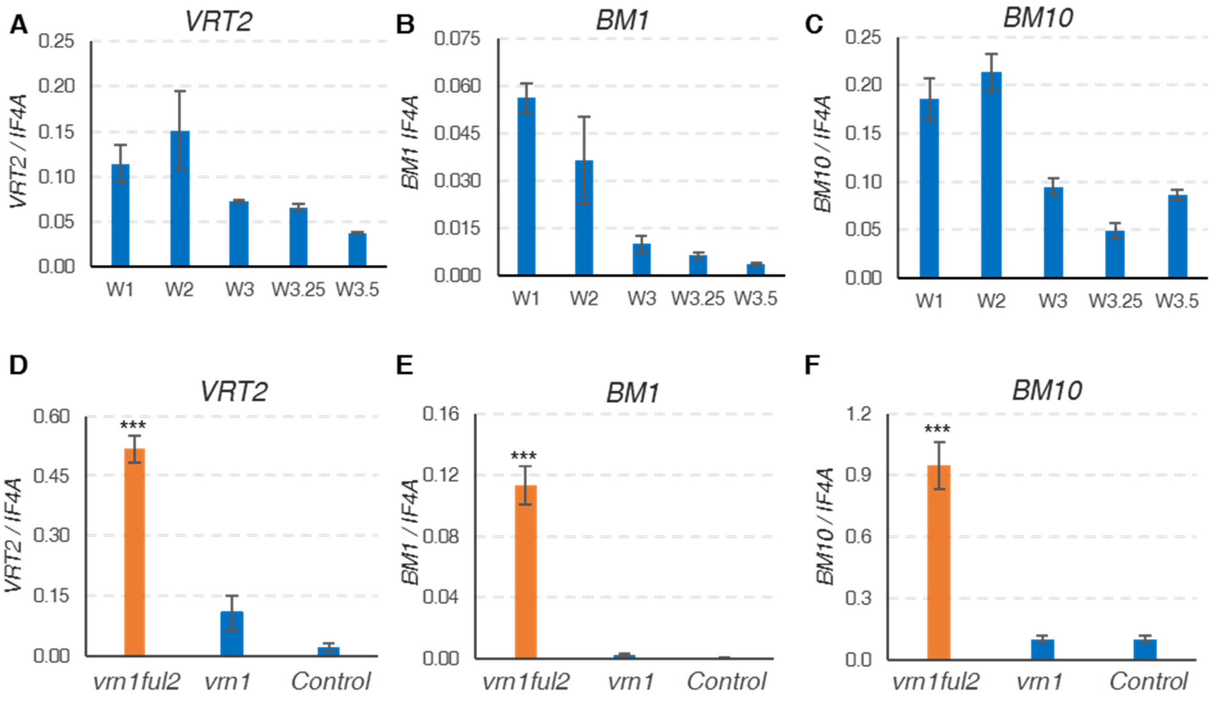

Fig. S7. Transcript levels of wheat *SVP*-like MADS-box genes *VRT2*, *BM1* and *BM10.* Transcript levels of (A & D) *VRT2* (*TraesCS7A02G175200* and *TraesCS7B02G080300*), (B & E) *BM1* (*TraesCS4B02G302600*), (C & F) *BM10* (*TraesCS6A02G313800* and *TraesCS6B02G343900*). (A-C) Normal spike development in wild type Kronos *(Vrn1Vrn2*) using the Waddington scale (W1.0= vegetative stage, W2.0 = double ridge stage, W3.0 = glume primordium present, W3.25 = lemma primordium present, W3.5 = and floret primordium present). (D-F) *vrn1ful2*-null, *vrn1*-null, and control at W3.5 (all in *vrn2*-null background). Transcript levels were calculated relative to the *INITIATION FACTOR 4A* (*IF4A*) as endogenous control using the ΔCt method. *** = $P < 0.0001$.

Development • Supplementary information

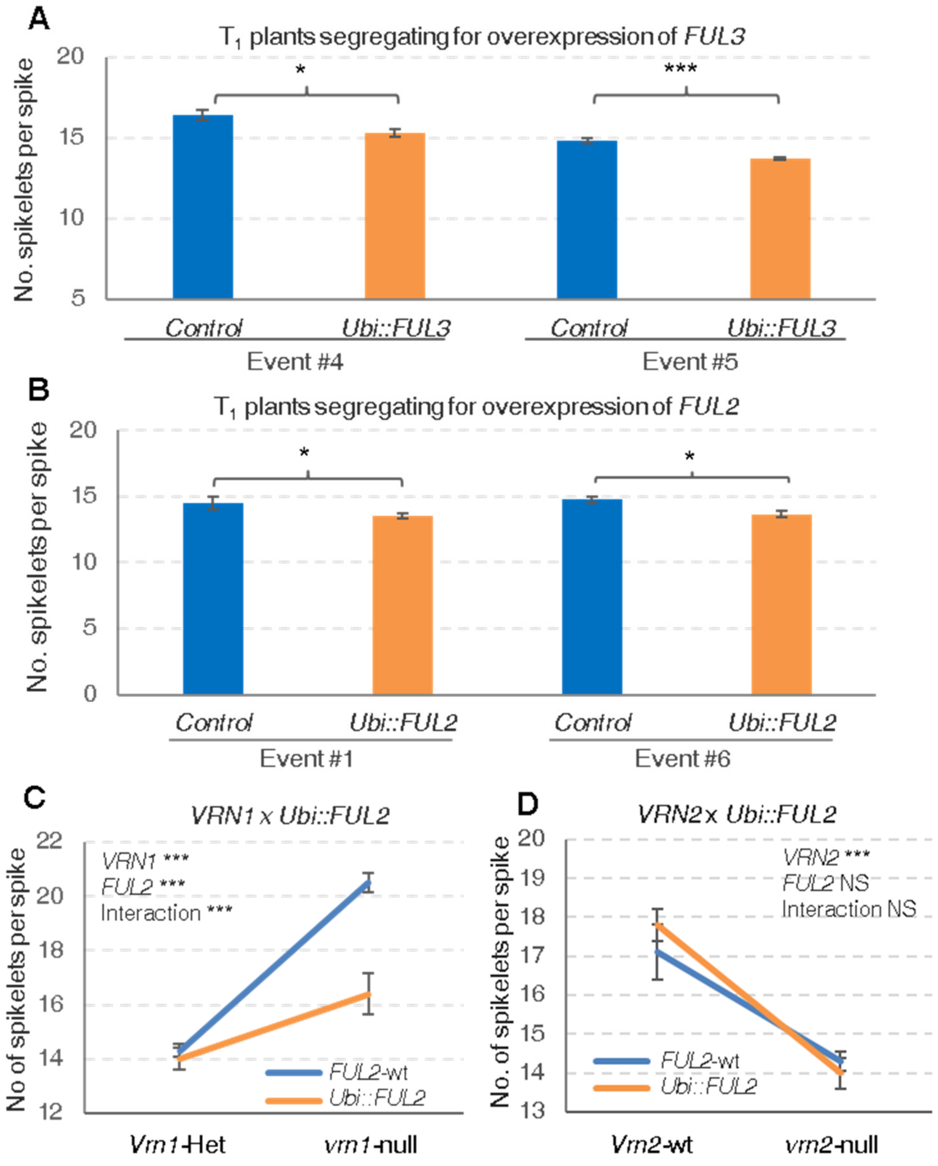

Fig. S8. Effect of the overexpression of *FUL2* and *FUL3* on the number of spikelets per spike. (A) Effect of *Ubi::FUL3* on the number of spikelets per spike in T_1 plants from two independent transgenic events: #4 (control n = 10, transgene n= 30) and #5 (control n = 9, transgene n= 31). (B) Effect of *Ubi::FUL2* on the number of spikelets per spike in T_1 plants from two independent transgenic events: #1 (control n = 4, transgene n= 23) and #6 (control n = 4, transgene n= 11). (C) F_2 plants segregating for *VRN1* and *FUL2* in a *vrn2*-null background (*Vrn1*-Het is heterozygous for both *VRN-A1* and *VRN-B1)*. (D) F_2 plants segregating for *VRN2* and *FUL2* in a *vrn1Vrn1* heterozygous background. (C-D) *P* values correspond to a 2 x 2 factorial ANOVA (N = 35). Error bars are SEM. * = $P < 0.05$, *** = $P < 0.0001$, NS = $P > 0.05$.

Development • Supplementary information

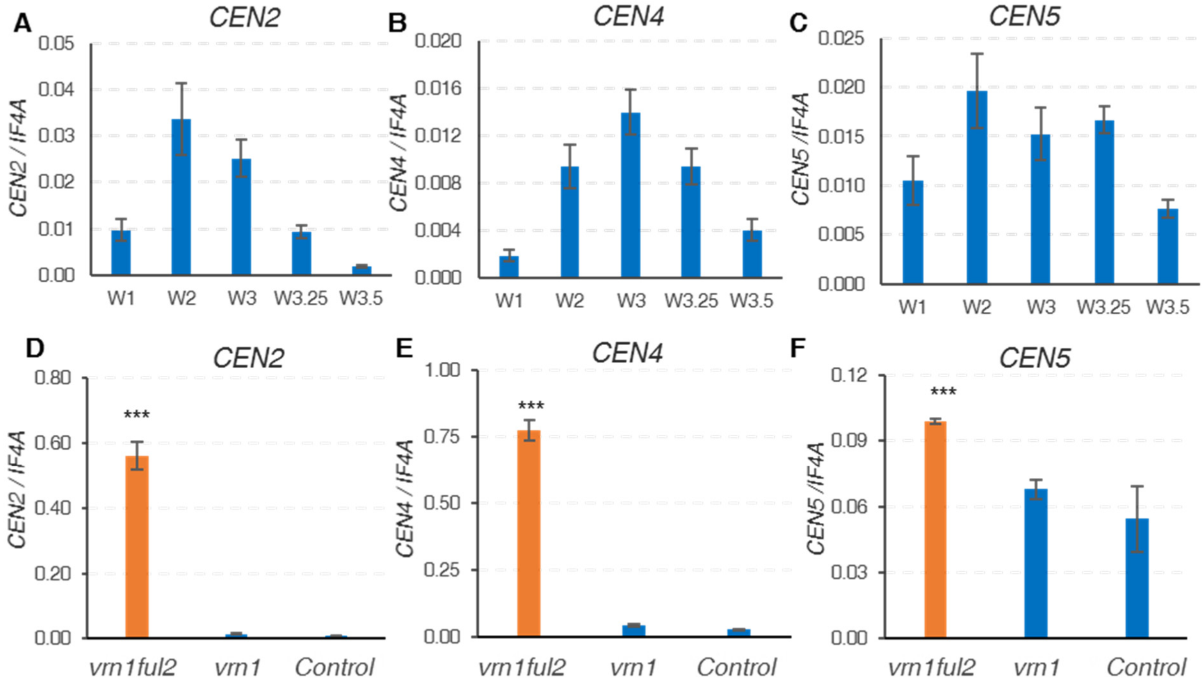

Fig. S9. Transcript levels of wheat *TFL1/CEN*-like genes *CEN2*, *CEN4* and *CEN5.* Relative expression levels of (A &D) *CEN2* (*TraesCSU02G202000* and *TraesCS2B02G310700*), (B & E) *CEN4* (*TraesCS4A02G409200* and *TraesCS4B02G307600*), (C & F) *CEN5* (*TraesCS5A02G128600* and *TraesCS5B02G127600*). (A-C) Normal spike development in wild type Kronos *(Vrn1Vrn2*) using the Waddington scale (W1.0= vegetative stage, W2.0 = double ridge stage, W3.0 = glume primordium present, W3.25 = lemma primordium present, W3.5 = and floret primordium present). (D-F) *vrn1ful2*-null, *vrn1*-null, and control at W3.5 (in *vrn2*-null background). Transcript levels were calculated relative to the *INITIATION FACTOR 4A* (*IF4A*) as endogenous control using the ΔCt method. *** = $P < 0.0001$.

Development • Supplementary information

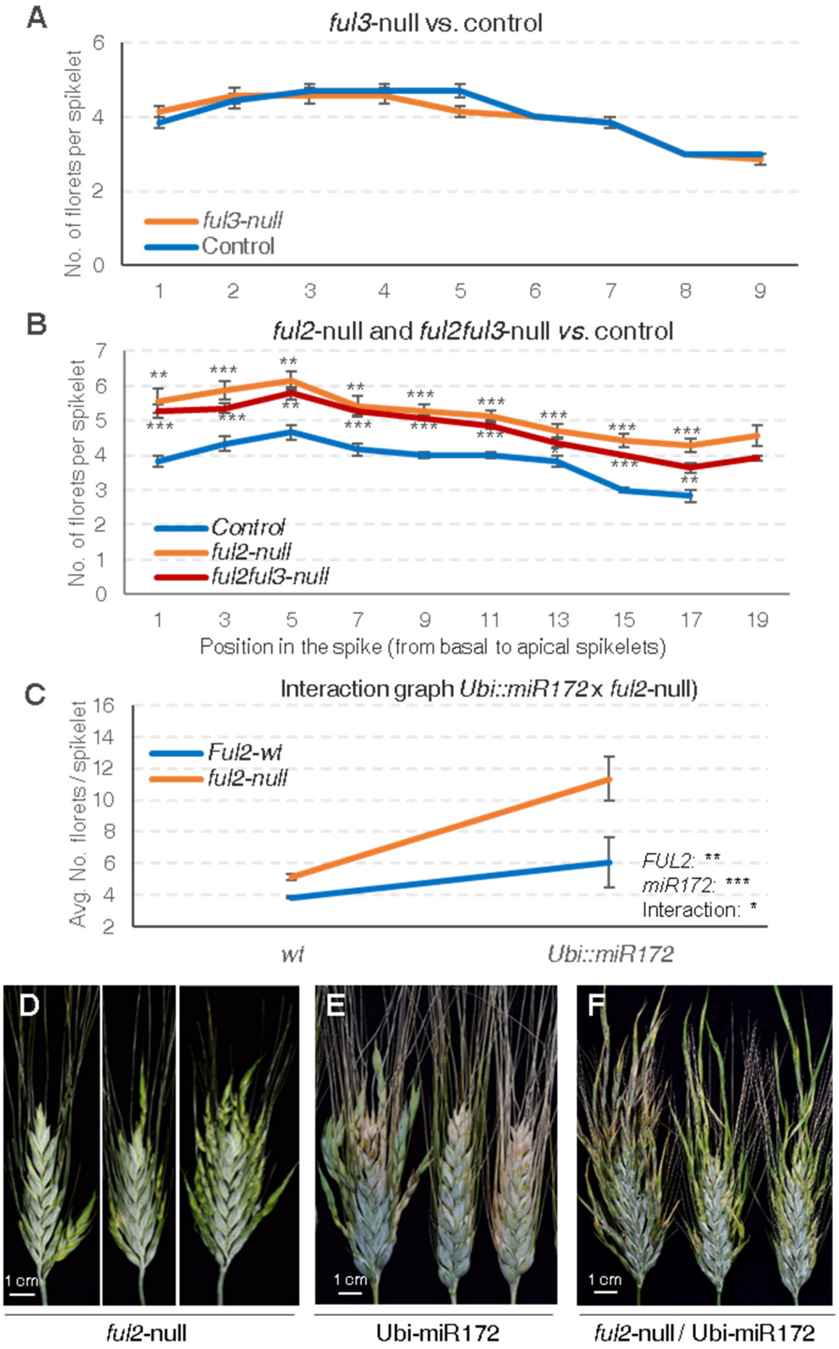

Fig. S10. Effect of *ful2*-null *ful3*-null and over-expression of *miR172* on the number of florets per spikelet. (A-B) Distribution of the number of florets per spikelet at different positions in the spike (florets from only one side of seven primary spikes were analyzed). (A) *ful3*-null *vs*. control. (B) *ful2*-null and *ful2ful3*-null *vs*. control (*P* values of each mutant versus the control, by position in the spike). (C) Interaction graph for floret number in F_2 plants from the cross *Ubi::miR172* x *ful2*-null. (D-F) Representative spikes showing heterogeneity in the distribution of spikelets with extra florets in (D) *ful2*-null, (E) *Ubi::miR172* and (F) *ful2*-null + *Ubi::miR172*. ** = $P < 0.01$, *** = $P < 0.001$, error bars are SEM.

Development • Supplementary information
